# Treatment success after rhinosurgery: an evaluation of subjective and objective parameters

**DOI:** 10.1007/s00405-021-06787-5

**Published:** 2021-04-03

**Authors:** M. M. Martin, K. Hauck, A. von Witzleben, J. Lindemann, M. O. Scheithauer, T. K. Hoffmann, F. Sommer

**Affiliations:** Dept. of Otorhinolaryngology and Head and Neck Surgery, Ulm University Medical Center, Frauensteige 12, 89075 Ulm, Germany

**Keywords:** Nasal obstruction, Nasal septal deviation, Nasal surgical procedures, Nose deformity, Rhinoplasty

## Abstract

**Purpose:**

Septal deviation and nose deformities are widely prevalent. As a consequence, patients may complain about difficulties in nasal breathing leading to a perception of diminished disease-specific quality of life. In a prospective randomized trial, we aimed to analyse the outcome of septoplasty (SPL) and septorhinoplasty (SRP) on patient satisfaction.

**Methods:**

Patients with functional indication for SPL (*n* = 19) or SRP (*n* = 54) were included and randomized for additional turbinoplasty. Preoperative clinical symptoms were collected with SNOT-20 GAV (Sinu-nasal outcome test-20—German adapted version) and NOSE^©^ (nasal obstruction symptom evaluation) questionnaires. The final evaluation of treatment success was performed 9 months after surgery with SNOT-20 GAV, NOSE^©^ and a self-established feedback questionnaire. Nasal breathing and obstruction were objectively measured with rhinomanometry and acoustic rhinometry [minimum cross-sectional area 2 (MCA2)].

**Results:**

Minimum cross-sectional area 2 was statistically improved compared to the pre-treatment value in SPL (*p* = 0.0004) and SRP (*p* = 0.0001). Regarding MCA2 values of matched patient groups, similar findings were detected (SPL: *p* = 0.0013, SRP: *p* < 0.0001).

Sinu-nasal outcome test-20 GAV and NOSE^©^ scores were significantly reduced after both surgical procedures (NOSE^©^: SPL: *p* < 0.0001, SRP: *p* < 0.0001; SNOT-20 GAV: SPL: *p* = 0.0068, SRP: *p* < 0.0001). Evaluation of patient satisfaction in a self-established feedback questionnaire revealed a motivation of 81% of patients to redo the surgery (SPL 13/16, SRP 34/42) and a notably general satisfaction of 86% for SPL and 80% for SRP.

**Conclusion:**

Rhinosurgery leads to quantitative better nasal breathing and increased disease-specific satisfaction. However, this study implies the importance of the right selection of patients and the correct indication of the surgical technique.

## Introduction

Septal deviation and deviated nose deformities are widely prevalent, with a rather high incidence [1]. Deviation of the inner and outer nose is not necessarily related to clinical symptoms. Some patients with these anatomical variations complain about difficult nasal breathing leading to a reduced quality of life (QOL).

According to the World Health Organization, QOL is defined as “individuals’ perception of their position in life in the context of the culture and value systems in which they live and in relation to their goals, expectations, standards and concerns” [2].

There are objective measurements available to assess nasal breathing, but their results are frequently not appropriate to the patient’s opinion [3–5]. The missing match of objective measurements and subjective feelings emphasizes the importance of an evaluation of patient-specific satisfaction. The benefit to the QOL of rhinosurgical procedures like septoplasty (SPL) or septorhinoplasty (SRP) remains controversial [6]. A large number of rhinosurgical methods can be chosen depending on the patients’ complaints and their anatomical pathologies. SPL and SRP belong to the most frequent surgical procedures in otorhinolaryngology. In Germany, in 2018, more than 100.000 SPL and 13.000 SRP procedures were undertaken [7]. While in SPL only, the nasal septum is corrected, in SRP, the correction of the external nose is part of the surgical challenge. Of course, indication criteria of these two surgical approaches differ and are based on the individual anatomical findings. Owing to ethical concerns, a comparison of both surgical strategies concerning patient satisfaction is difficult. Nevertheless, a differentiation of patient satisfaction dependent on the performed surgical procedure should be given attention. In our previously published study, the randomization was done for turbinoplasty in the first place, to see how this additional surgical approach can change the patient’s satisfaction and nasal breathing benefits. Intriguingly, this study revealed no significant changes attributed by the turbinoplasty for SPL and SRP [8].

Especially due to the predominantly subjective complaints of the patients, evaluation of disease-specific quality of life before and after rhinosurgery is a particular interest.

In a prospective randomized trial, we aimed to evaluate clinical outcome and patient’s satisfaction before and after SPL and SRP. We assessed subjective and objective parameters before and after rhinosurgery and correlated it with the accomplished surgery.

## Materials and methods

The study was authorized by a local ethics committee (Ethics Approval Number: 326/15). Anonymization was established via an identity code. Calculations were done using Microsoft Excel (Version 16.31). Statistical analyses and graphs were performed using SPSS Statistics 25 and Graph Pad Prism (Version 8.4.2). Mann–Whitney test was used for unpaired and Wilcoxon test for paired analyses. The Spearman’s coefficient was calculated for the correlation analysis.

### Patient cohort

In this prospective monocentric controlled trial, we included 73 patients with nasal obstruction and indication for SPL or SRP (patient characteristics in Table 1). In the SRP group, only patients with functional complaints due to nasal obstruction were included. Patients were randomized by additional turbinoplasty or no additional turbinoplasty in a blinded fashion. In the SPL group, 19 patients were included while the SRP group consisted of 54 patients.

**Table 1:** Patient characteristics

		All (*n*)	%	SPL (*n*)	%	SRP (*n*)	%
All		73	100	19	26	54	74
Gender	Male	47	64	15	79*	32	59*
	Female	26	36	4	21*	22	41*
Age (years)	Mean (range)	30.16 (18–56)		31.89 (20–56)		29.56 (18–54)	
Turbinoplasty	Yes	37	51	9	47*	28	52*
	No	36	49	10	53*	26	48*

*SPL* septoplasty, *SRP* septorhinoplasty

^*^Percentages calculated for patient number in SPL and SRP group, respectively

Patients with an age under 18 and over 60 years, previous nasal surgeries, severe allergic symptoms, obstructive sleep apnea, planned paranasal sinus surgery or smokers with more than one cigarette pack per day were excluded.

### Rhinometry

Objective measurement of nasal airflow with pre and postoperative rhinomanometry using the Rhino4000M (Homoth Medizinelektronik GmbH and Co. KG, Kaltenkirchen, Germany) was performed. The nasal airflow was measured using the inspiratory flow in ml/s at a pressure of 150 Pa as a total of both nasal sides without decongestion.

Acoustic rhinometry was performed with the RhinoSys (Happersberger otopront GmbH, Hohenstein, Germany) without decongestion. The minimal cross-sectional area 2 (MCA2) in cm^2^ was measured by acoustic reflections.

### Subjective values for disease-specific QOL

Preoperative clinical symptoms were evaluated using SNOT-20 GAV [9] and NOSE^©^ questionnaire [10, 11]. SNOT-20 GAV is a German adapted version of the Sino-Nasal Outcome Test 20 [9] and is a validated instrument to assess health-related QOL in patients with chronic rhinosinusitis. SNOT-20 GAV questionnaire elevates a total score and consists of three sub-scores: primary nasal symptoms (PNS), secondary rhinogenic symptoms (SRS) and general quality of life (GQL). NOSE^©^ is an acronym for nasal obstruction symptom evaluation and is a validated questionnaire consisting of 5 categories. Both questionnaires are reported on a scale from 0 (no symptoms) to 100 (severe symptoms). A final evaluation of patient satisfaction was done 9 months after surgery, containing SNOT-20 GAV, NOSE^©^ and a self-established feedback questionnaire. The self-established feedback questionnaire requested the patient´s general satisfaction and the willingness to do the surgery again. 53 of 74 (71%) patients could be consulted to complete all questionnaires.

## Results

### Reduction of nasal obstruction after SPL and SRP

**Fig. 1:**
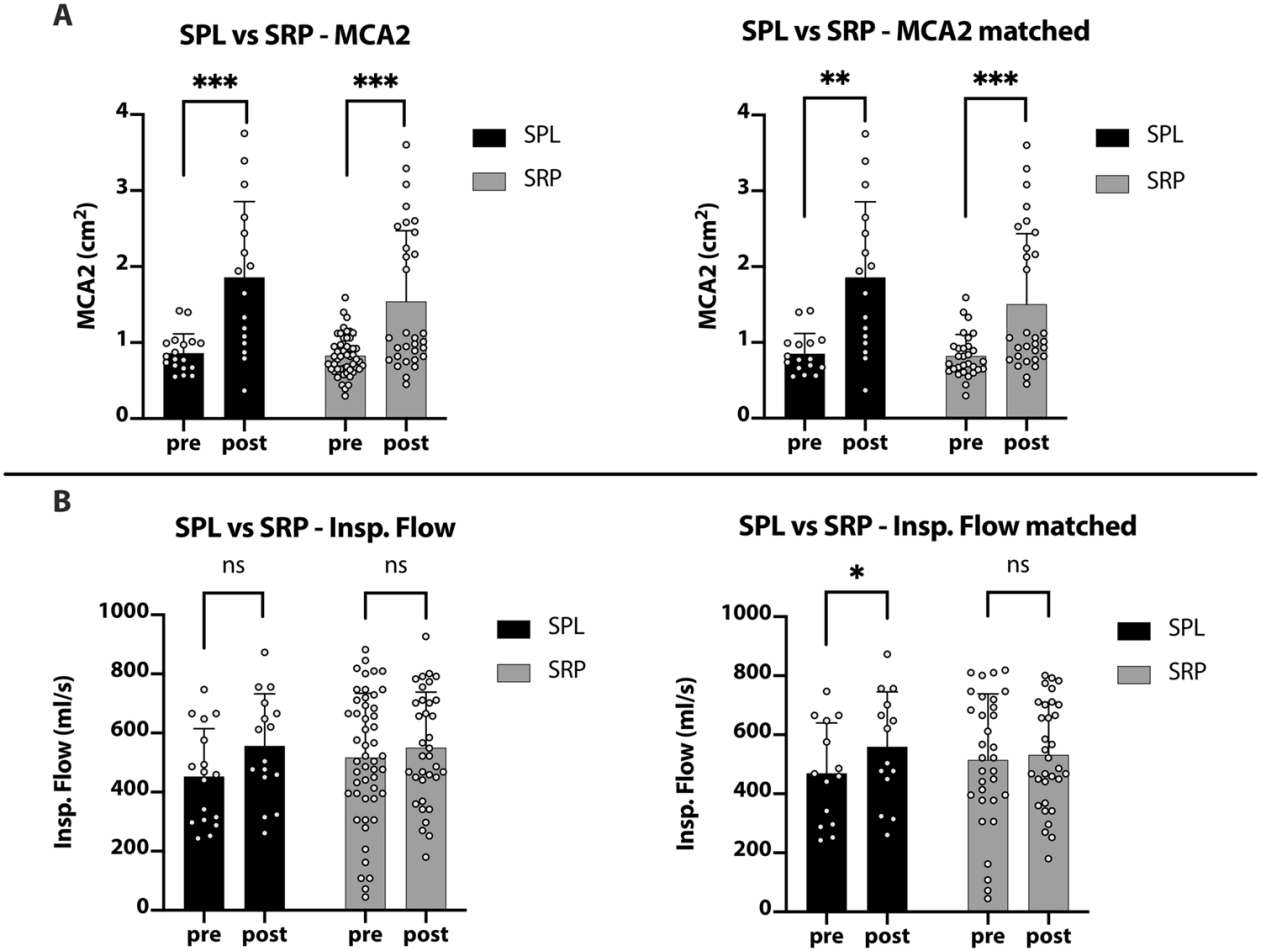
**a**: Bar plot showing pre and postoperative acoustic rhinometry (MCA2) values in SPL (pre: *n* = 18, post: *n* = 16) and SRP (pre: *n* = 50, post: *n* = 31) using Mann–Whitney-Test and pairwise comparison of SPL (pairs: *n* = 16) and SRP (pairs: *n* = 30) using Wilcoxon matched test. **b** Bar plot showing rhinomanometry (inspiratory flow rate) values in SPL (pre: *n* = 78, post: *n* = 16) and SRP (pre: *n* = 50, post: *n* = 35) using Mann–Whitney-Test and pairwise comparison of SPL (pairs: *n* = 14) and SRP (pairs: *n* = 32) using Wilcoxon matched test

Nasal obstruction was measured using acoustic rhinometry by collecting MCA2. The values were compared before and after surgery and graphed in Fig. 1a. In the SRP group, one case had a postoperative MCA2 value of 6.0cm^2^, which was considered to be an outlier and was therefore excluded in further analysis.

The mean MCA2 was determined with 0.9cm^2^ [standard deviation (SD): 0.3cm^2^, *n* = 18] preoperatively vs. 1.9cm^2^ (SD 1.0cm^2^, *n* = 16) postoperatively in the SPL group and 0.8cm^2^ (SD 0.3cm^2^, *n* = 50) preoperatively vs. 1.5cm^2^ (SD 0.9cm^2^, *n* = 32) postoperatively in the SRP group. When comparing the SPL and SRP groups at the preoperative and separately at the postoperative point of time, no statistical difference was found.

Considering pre and postoperative MCA2 for both surgical approaches, a significant increase could be shown after SPL (*p* = 0.0004) and SRP (*p* = 0.0003). Respecting paired pre- and postoperative values comparable results were observed (SPL *p* = 0.0013, SRP *p* = 0.0002).

### Inspiratory flow rates increased

The mean inspiratory flow rate was calculated with 452.5 ml/s (SD 162.5 ml/s) (*n* = 17) preoperatively vs. 556.3 ml/s (SD 176.0 ml/s) (*n* = 16) postoperatively in the SPL group and 517.1 ml/s (SD 217.6 ml/s) (*n* = 50) preoperatively vs. 550.8 ml/s (SD 187.4 ml/s) (*n* = 35) postoperatively in the SRP group (Fig. 1b).

The inspiratory flow rates did not significantly differ respecting pre and postoperative values of the therapy groups (pre vs postoperative SPL: *p* = 0.0887, SRP: *p* = 0.6550). However, the paired analysis revealed a statistically significant increase in the postoperative flow rate in the SPL patient group [pre vs. postoperative SPL: *p* = 0.0234 (*n* = 14)].

### General Satisfaction in self-established feedback questionnaire

The general satisfaction after both rhinosurgical approaches was high. In the postoperative feedback questionnaire 9 months after surgery, 86% (12/14) of SPL patients were satisfied, while 80% (35/44) of SRP patients were confident with the outcome. 81% of the patients would have agreed to do the surgery again, 13/16 of patients after SPL and 34/42 of patients after SRP.

### ***NOSE***^***©***^*** and SNOT-20 GAV questionnaires***

**Fig. 2:**
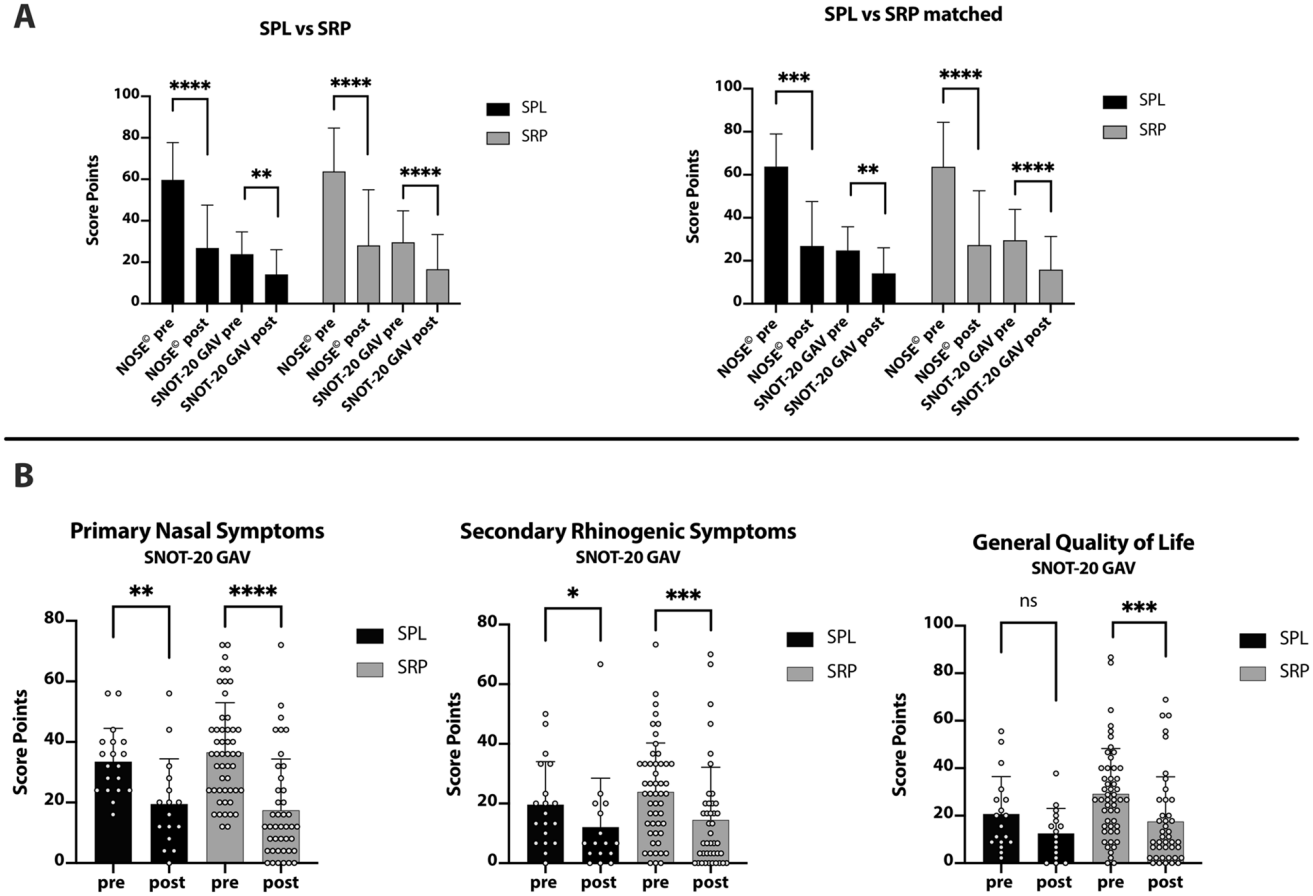
**a** Bar plot graphing NOSE ^©^ values: pre vs post SPL: *p* < 0.0001, SRP: *p* < 0.0001 and SNOT-20 GAV: pre vs post SPL: *p* = 0.0068, SRP: *p* < 0.0001 (Mann–Whitney-Test); and paired NOSE^©^ values: pre vs post SPL: *p* = 0.0002, SRP: *p* < 0.0001; SNOT-20 GAV values: pre vs post SPL: *p* = 0.0038, SRP: *p* < 0.0001 (Wilcoxon matched test). **b** Bar plots displaying the sub-scores of SNOT-20 GAV for primary nasal symptoms (pre vs postoperative: SPL: *p* = 0.0015; SRP: *p* < 0.0001), secondary rhinogenic symptoms (pre vs postoperative: SPL: *p* = 0.04; SRP: *p* = 0.0008) and general quality of life (pre vs postoperative: SPL: *p* = 0.1185; SRP: *p* = 0.0005)

The mean total score of NOSE^©^ was calculated with 59.7 points (SD 18.0 points) (*n* = 19) preoperatively vs. 26.9 points (SD 20.7 points) (*n* = 16) postoperatively in the SPL group and 63.8 points (SD 20.9 points) (*n* = 52) preoperatively vs. 28.1 points (SD 26.9 points) (*n* = 42) postoperatively in the SRP group (Fig. 2a).

The mean total score of SNOT-20 GAV was calculated with 23.8 points (SD 10.8 points) (*n* = 18) preoperatively vs. 14.1 points (SD 11.9 points) (*n* = 16) postoperatively in the SPL group and 29.6 points (SD 15.2 points) (*n* = 48) preoperatively vs. 16.6 points (SD 16.8 points) (*n* = 42) postoperatively in the SRP group (Fig. 2a).

Comparing pre and postoperative overall score of NOSE^©^-questionnaires, both surgical approaches showed a significant decrease in symptoms (SPL pre vs. postoperative: *p* < 0.0001, SRP pre vs postoperative: *p* < 0.0001). Comparable results were demonstrated for overall score of SNOT-20 GAV (SPL pre vs postoperative: *p* = 0.0068, SRP pre vs postoperative: *p* < 0.0001). In a pairwise analysis, similar results were obtained [NOSE^©^: SPL pre vs. postoperative: *p* = 0.0002 (*n* = 16), SRP pre vs postoperative: *p* < 0.0001 (*n* = 40); SNOT-20 GAV: SPL pre vs. postoperative: *p* = 0.0038 (*n* = 16), SRP pre vs postoperative: *p* < 0.0001 (*n* = 38)] (Fig. 2a). Pre and postoperative values of both treatment groups were not statistically different.

Especially the primary nasal symptoms within SNOT-20 GAV could be reduced by both surgical procedures (SPL pre vs postoperative: *p* = 0.0015, SRP pre vs postoperative: *p* < 0.0001) (Fig. 2b). Additionally, secondary rhinogenic symptoms within SNOT-20 GAV were significantly diminished (SPL pre vs postoperative: *p* = 0.04, SRP pre vs postoperative: *p* = 0.0008) (Fig. 2b). Considering general quality of life within SNOT-20 GAV, a significant improvement could be revealed only in SRP group, in SPL group, differences were not significant (pre vs postoperative: SPL *p* = 0.1185, SRP: *p* = 0.0005) (Fig. 2b).

**Fig. 3:**
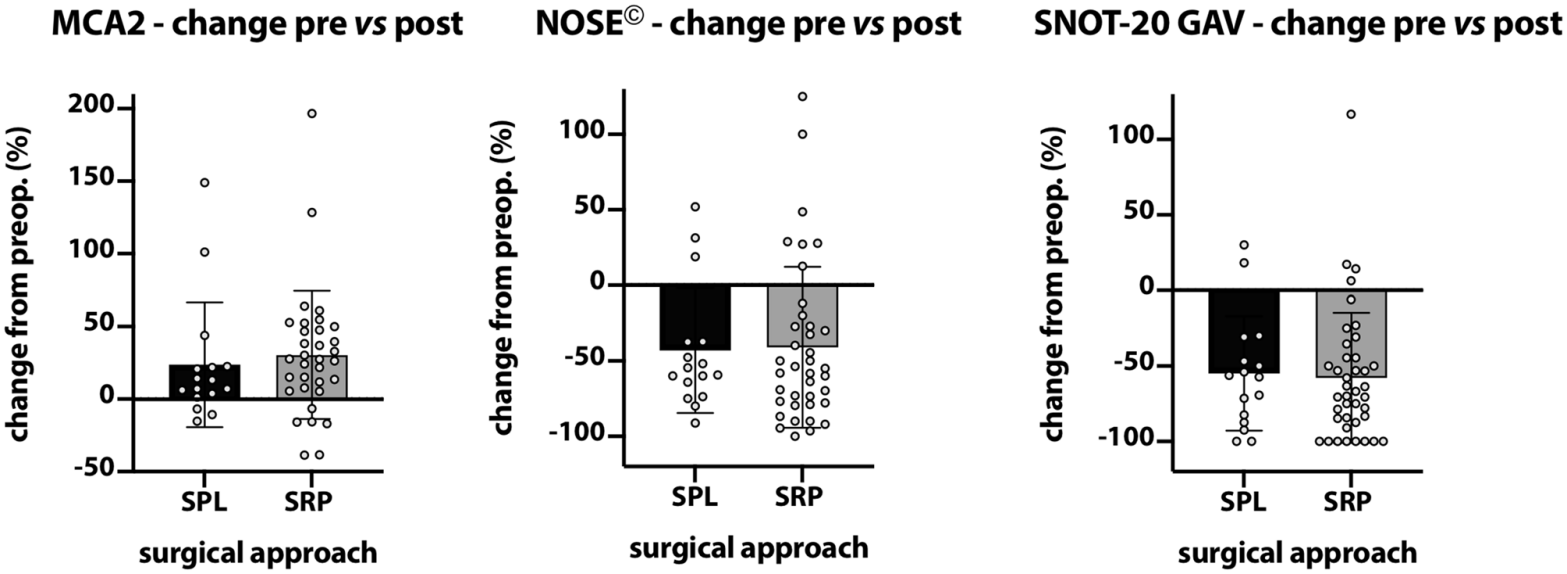
Bar plot showing the changes of MCA2, SNOT-20 GAV and NOSE ^©^ values normalized to the presurgical individual values in percent

The change of MCA2-values showed a noticeable increase comparing pre- and postoperative results (Fig. 3). The changes in SNOT-20 GAV and NOSE^©^ questionnaires illustrated a remarkable decrease in point values in both approaches (Fig. 3). However, this summarizing analysis underpins that no statistical differences were detected between both surgical procedures.

**Fig. 4:**
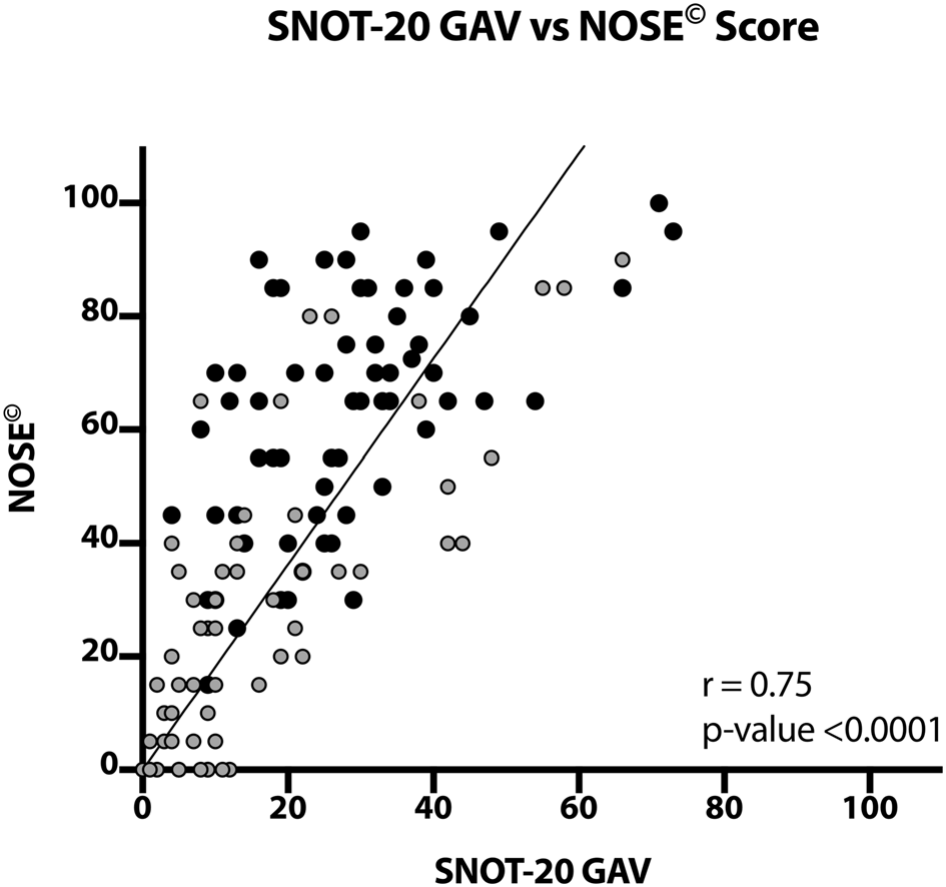
Scatter plot displaying all SNOT-20 GAV and NOSE ^©^ values with a statistically significant positive Spearman correlation (*p* < 0.0001, *r* = 0.75) between those two questionnaires. Black dots: presurgical values; Grey dots: postsurgical values

The correlation between the pre and postsurgical questionnaire values is graphed in Fig. 4. The Spearman’s analysis disclosed a significant correlation (*p* < 0.0001) of SNOT-20 GAV values with the NOSE^©^ values (*r* = 0.75). It becomes apparent, that the data points are shifted towards the left lower corner after surgical treatment.

## Discussion

Anatomy of the nasal septum is complex and there is no standardized classification regarding the grade and type of deviation. There are multiple reasons for nasal obstruction, which often occur together. Individual perception of complaints due to septal deviation or deviated outer nose is very different. Due to diverse anatomical conditions and various impairments of complaints, there is no standard staging system for the indication of SPL or SRP. For this reason, indication for surgery is always an individual decision. Nevertheless, there are many borderline cases in which a deviated nose has only a minor influence on nasal breathing and an SPL alone can bring a remarkable benefit. Conversely, there are constellations with a septal deviation in combination with subluxation due to a deviation of the external nose, where rhinoplasty is much more appropriate to achieve treatment success. Therapeutic success can be defined on the one hand as an objectively measured improvement of nasal breathing and, on the other hand, as an increase in the subjective patient’s QOL.

The latter has increasing relevance in modern medicine as QOL assessment allows an individual estimation of therapy success. Additionally, evidence-based medicine plays an important role in rhinosurgery although the subjective benefit of surgical procedures is difficult to measure [12]. This needs high-quality studies to prove the effectiveness of surgical therapies. Prospective randomized controlled trials can address these conditions, but unfortunately, they are rare in surgical therapies due to their challenging design. Thus, there is little known about the effect of rhinosurgical procedures on disease-specific perception of complaints. In our study, we aimed to investigate objective and subjective parameters of disease-specific QOL to estimate the change in subjective perception of complaints after rhinosurgery. Furthermore, we wanted to investigate whether patients with SRP benefit more from the surgical intervention than patients with SPL.

As described in our recently published study, turbinoplasty in addition to SPL or SRP does not have a significant impact on subjective as well as objective values [8]. A study, randomizing patients between SPL and SRP would be of high interest. However, randomization of patients between SPL and SRP is mainly not possible due to ethical reasons as well as different anatomical preconditions of each patient. Nevertheless, there are borderline cases in which SPL, as well as SRP, is possible to address the complaints of a patient and to improve nasal geometry.

Regarding objective rhinological data as acoustic rhinometry (MCA2), significant changes between pre- and postoperative values in SPL and SRP were obvious. Interestingly, the postoperative inspiratory flow did not significantly increase in both treatment groups in comparison to preoperative results. In former publications, authors pointed out, that subjective patient’s complaints are not always consistent with objective data [13, 14]. Nevertheless, there is evidence that SPL improves objective outcome [3, 15].

However, these objective tests underly limitations. Especially rhinomanometry highly depends on cooperation of the patient. But no other diagnostics are available and therefore remain a standard in clinical practice.

Nasal surgery is primarily focusing on functional aspects, but also on aesthetic problems caused by deformities of the external nose. Rhinosurgery often addresses symptoms that are influenced by subjective perception of associated complaints and psychosocial factors may have a high impact on postoperative satisfaction.

A validated instrument to evaluate the symptom “nasal obstruction” is the NOSE^©^ questionnaire [10, 11]. The NOSE^©^ scores revealed significant fewer symptoms for SPL and SRP. SNOT-20 GAV is a validated score to assess health-related QOL in patients with chronic rhinosinusitis. In the SNOT-20 GAV questionnaire, similar effects could be detected for SRP regarding overall score and sub-scores. In the SPL cohort, overall score and sub-scores significantly decreased, except for the sub-score “GQOL”. One could presume that the postoperative benefit for disease-specific QOL for SPL is not as high as for SRP. However, general satisfaction in the self-established feedback questionnaire was remarkably high.

Due to the lack of tools to acquire disease-specific QOL, rhinological questionnaires are used as an instrument to estimate the patients’ satisfaction after rhinosurgery, even though not being designed for this purpose.

Due to the increasing questioning of established therapies, studies of higher qualities comparing surgical procedures are necessary. Of course, due to the reasons mentioned above, these cannot be set up without problems. However, the present study shows that both, SPL and SRP provide remarkable subjective and objective results. Of course, SPL cannot replace SRP in general, but our results show that despite increasing discussion about the usefulness of SPL [16], its results are comparable to those of SRP when patient selection is correct.

## Conclusion

Septoplasty and SRP lead to decreased symptom scores in validated questionnaires (NOSE^©^/SNOT-20 GAV) and to partially improved nasal breathing assessed by objective tests as well. Hence, investigating functional aspects using the NOSE^©^ questionnaire and determining nasal symptoms as well as disease-specific QOL using the SNOT-20 GAV questionnaire revealed a postoperative advantage in both cohorts. However, postoperative outcome and assessment of the success of rhinosurgery are highly dependent on the patient´s opinion and subjective perception of complaints. Identification of factors that can predict postoperative success is one of the main aims of future studies.
